# Editorial: Big data and artificial intelligence in ophthalmology

**DOI:** 10.3389/fmed.2023.1145522

**Published:** 2023-02-14

**Authors:** Sahil Thakur, Tyler Hyungtaek Rim, Darren S. J. Ting, Yi-Ting Hsieh, Tae-im Kim

**Affiliations:** ^1^Department of Ocular Epidemiology, Singapore Eye Research Institute, Singapore, Singapore; ^2^Mediwhale Inc., Seoul, Republic of Korea; ^3^Academic Unit of Ophthalmology, Institute of Inflammation and Ageing, University of Birmingham, Birmingham, United Kingdom; ^4^Birmingham and Midland Eye Centre, Birmingham, United Kingdom; ^5^Academic Ophthalmology, School of Medicine, University of Nottingham, Nottingham, United Kingdom; ^6^Department of Ophthalmology, National Taiwan University Hospital, Taipei, Taiwan; ^7^Department of Ophthalmology, College of Medicine, National Taiwan University, Taipei, Taiwan; ^8^Department of Ophthalmology, The Institute of Vision Research, Yonsei University College of Medicine, Seoul, Republic of Korea; ^9^Department of Ophthalmology, Corneal Dystrophy Research Institute, Yonsei University College of Medicine, Seoul, Republic of Korea

**Keywords:** big data, ophthalmology, artificial intelligence, deep learning, machine learning

Big Data and Artificial Intelligence (AI) are rapidly transforming modern healthcare. The combination of these technologies allows for the collection, analysis, and utilization of large amounts of healthcare data in ways that were previously not possible. While there are several challenges to overcome, the potential benefits of using these technologies are significant and include improved patient outcomes, efficient and effective healthcare delivery, and potential of improving access and affordability of healthcare interventions. Ophthalmology is a field that generates a large amount of healthcare data, including images of the retina, cornea, and other eye structures (1). The widespread use of electronic health records has also led to an increase in the amount of patient data available for analysis (2, 3). Thus, ophthalmology is a great medical subspeciality for applications that can utilize big data and AI.

This Research Topic focuses on the utility and the potential of big data and AI in ophthalmology. Authors from a broad spectrum of vision science and ophthalmology associated specialties from several countries, have contributed to this Research Topic. They have highlighted novel uses of large datasets, introduced new perspectives, and have reported AI algorithms with immense translational potential. In this editorial we provide a thematic overview of the exciting and diverse content covered under this Research Topic.

## 1. Publication trends

Yang et al. have performed a bibliometric analysis of the publication trends in AI in retina from 2012 to 2022 and report interesting findings. Countries like US and China have been leading the research output with maximum number of publications (US:171, China: 149), citations (US:2466, China: 1401), and H-index (US:28, China: 20). However, several issues such as lack of real-world testing of algorithms, meaningful economic impact assessment, use of multimodal imaging data for algorithm development and ethical, regulatory, and legal complexities associated with dataset curation need to be addressed by the researchers. Additionally, adequate representation of different populations in training datasets and algorithm generalizability are other areas of concern that need attention.

## 2. Anterior segment

In this Research Topic we observe the utility of big data to answer perplexing clinical problems. Kwak et al. used the KNHIS-Senior database (*n* = 558,147) to demonstrate that the rate or risk of surgical complications of cataract surgery did not change with tamsulosin use in the Korean elderly population. These findings contradict conventional understanding that intraoperative floppy iris syndrome (IFIS) is frequent in patients taking tamsulosin and can cause significant perioperative or postoperative complications during cataract surgery (4). Though the authors were cautious in interpreting their findings and attributed their results to careful surgeon's effort to respond to perioperative complications and advances in surgical equipment, the big data driven approach of the study shows how conventional observational “wisdom” may sometimes not hold true when tested against benchmarks of real world “evidence.” Another study by Ahn et al. (a) demonstrated the value of big data by showing that surgically induced astigmatism (SIA) was higher in the femtosecond assisted cataract surgery + arcuate keratotomy group than the conventional phacoemulsification group (0.886 vs. 0.631 *p* < 0.001). The overcorrection ratios were also higher in the in the femtosecond group (58.9%) vs. the conventional group (48.8%). Though the femtosecond laser was effective when target induced astigmatism (TIA) values are greater than 0.75 D, overcorrection in patients with a lower degree of astigmatism and the angle of error in patients with higher astigmatism may lead to higher postoperative corneal astigmatism. Further research is thus needed to understand the factors that affect astigmatism in femtosecond laser assisted cataract surgery.

Ahn et al. (b) also demonstrated that multi-source ASOCT images can be used for estimating preoperative best corrected visual acuity (BCVA). This AI biomarker can be used as a surrogate for cataract grade as well. The authors also reported that in the subgroup which had an absolute error (AE) ≥ 0.1, subjects had significant vision impairing disease like macular disease/glaucoma or another optic neuropathy. Thus, a more intuitive approach would be to use both anterior and posterior segment imaging to formulate an algorithm that provides an estimate of both pre and postoperative BCVA. Surgeons and patients would then be able to effectively manage expectations and deliver satisfying outcomes.

Qian et al. showed that anterior chamber depth (ACD) can be predicted using smartphone captured images of the anterior segment. The MAE reported by the authors was 0.16 ± 0.13 mm, and *R*^2^ between the predicted and measured ACD was 0.40. The central corneal region was highlighted in the saliency maps indicating that the predicted ACD was correlated with the clinically used site for ACD measurement. Such algorithms that utilize easily available consumer technology for image capture and subsequently can predict important ocular biomarkers have potential for rapid deployment in the real world.

Ahn et al. (c) also demonstrated the possibility of automating hospital workflows by predicting pupil dilation based on medical interview and basic eye examinations data. Using a large well-curated dataset of 56,811 patients over a period of 3 years, the authors demonstrated a sensitivity of 94.2–75.7% and specificity of 96.2–96% for predicting the need of a pupil dilation test only based on basic clinical information. The authors identified that asymptomatic lesions however led to reduced performance of the model, though it is still interesting to see how big data and innovative AI algorithms may improve and automate hospital and clinic workflows.

## 3. Posterior segment

Lin et al. showed that OCT images could be used to infer VA information using a deep learning algorithm in patients with diabetic macular edema (DME). Traditionally VA is documented using chart based methods that are prone to subjectivity and depend on chart quality and illumination. AI based methods for VA estimation can be thus used for subjects with poor cooperation and provide surrogate functional vision endpoints for monitoring.

Lu et al. demonstrated prediction of axial length classes using choroidal thickness measures from 2D OCT images. The model however requires 6 point choroidal thickness measurement and variables like age, gender, height, and weight for axial length classification. Development of future AI models that can predict axial length without additional demographic information can be explored using larger and multimodal datasets.

Wang et al. demonstrate a deep learning model for DME classification with AUC range of 98.1–95.2%, sensitivity of 96.4–87.4%, and specificity of 90.2–90.1% in three large datasets. The model is novel as it can localize hard exudates along with anatomical landmarks. Diabetic retinopathy (DR) and its associated complications offer several opportunities for AI based algorithm development and potential use due to ease of retinal image capture using portable fundus cameras, well-defined disease labels and high disease prevalence.

In another study using big data from the Korean NHIS-Senior database, Kim et al. showed that in elderly patients with retinal diseases, the vitrectomy group showed the lower mortality from pulmonary causes when compared to those without vitrectomy. The associations were different based on underlying vitreoretinal disease: higher risk of all-cause mortality and vascular causes in patient subgroup with retinal vascular diseases and lower risk of all-cause mortality, vascular causes, and pulmonary causes in those with macular diseases. Such serendipitous results are difficult to explain as in this study a greater proportion of the patients with macular diseases who underwent vitrectomy were current or ex-smokers who regularly consumed alcohol. However, such results inspire discussion and future research into the impact of surgical interventions for ocular disease on patient mortality.

## 4. APPRAISE survey

Gunasekeran et al. conducted the Acceptance and Perception of Artificial Intelligence Usability in Eye Care (APPRAISE) survey to evaluate the global perspective of ophthalmologists (*n* = 1176) regarding AI, focusing on four major eye conditions, namely DR, age related macular degeneration, glaucoma, and cataract. This survey highlighted that most respondents (80.9%) believed that the pandemic had played an important role in the willingness to adopt AI tools due to global focus on teleophthalmology and digital solutions for patient care while lockdowns were being implemented. While the goal of AI developers is to provide comprehensive AI solutions for patient care, ophthalmologists are more willing to use AI as clinical assistive tools (88.1%), when compared to clinical decision support tools (78.8%) or diagnostic tools (64.5%). The survey also highlighted the perceived advantages of AI based tools in patient screening (94.5%), improved access (84.7%), affordability (61.9%), quality (69.4%), targeted referrals (87.1%), and reduction of monotonous work (82.7%). Some potential disadvantages of AI like concerns over medical liability for errors (72.5%) and data security/privacy concerns (64.9%) were also mentioned. While AI is often mentioned as a threat to jobs in mainstream media, most survey responders were confident their roles will not be replaced (68.2%). The survey thus provides a comprehensive insight into the current perspective of ophthalmologists regarding AI based tools. The results can be utilized by all stakeholders to facilitate effective communication and formulate targeted interventions to address barriers that hamper development, adoption, and use of AI tools in ophthalmology.

## 5. Myopia and future trends

Zhang X. et al. report alarming projections of myopia affecting 8.57 million children (7–12 years) and 15.77 million adolescents (13–18 years) by the year 2050 in eastern China. Simple low cost interventions like outdoor activities, frame glasses and eye exercises have high utilization prevalence and can significantly reduce the burden of myopia. AI can also be used as a tool for myopia detection, monitoring and management. In their review Zhang C. et al., highlighted the potential uses of AI in addressing myopia holistically. With the advent of virtual reality (VR) and augmented reality (AR) into consumer realm, there are also opportunities to also develop intelligent digital tools that can aid in behavioral interventions for myopia control. However early detection of myopia and its complications followed by timely therapeutic interventions remain critical for myopia management.

## 6. Conclusion

This Research Topic has a diverse array of publications that cover a spectrum of topics dealing with the use of big data and applications of AI in ophthalmology. The ideas, algorithms and perspectives discussed and published by the researchers in this topic have potential for considerable impact on shaping the future landscape of ophthalmology. Collaborative efforts built on these foundations can help in translating these innovations across the frontiers of ophthalmology and medicine for effective patient care and welfare.

## Author contributions

ST contributed to conception and design of the study and wrote the first draft of the manuscript. All authors contributed to manuscript revision, read, and approved the submitted version.
